# Dynamic Concatenation of Quantum Error Correction in Integrated Quantum Computing Architecture

**DOI:** 10.1038/s41598-019-39439-0

**Published:** 2019-03-01

**Authors:** Ilkwon Sohn, Jeongho Bang, Jun Heo

**Affiliations:** 1 School of Electrical Engineering, Korea University, Seoul, Korea; 2 School of Computational Sciences, Korea Institute for Advanced Study, Seoul, 02455 Korea; 3 Advanced KREONET Center, Korea Institute of Science and Technology Information, Daejeon, Korea

## Abstract

Resource overhead problem caused by concatenation in quantum error correction (QEC) is of significant importance for the realization of fault-tolerant quantum computation (FTQC). To attack this problem, we propose a novel scheme by considering integrated FTQC architecture where the concatenation level is controlled *dynamically*; i.e., less (or more) concatenation levels are imposed by good (or poor) performance gates—we call this scheme “dynamic concatenation” in this sense. Such a dynamic concatenation is realizable in an integrated structure of FTQC, as the information of the concatenation can be communicated between classical system elements (e.g., compiler and system organizer) and the logical qubits in real-time. We derive the effective lower and upper bounds of the length of gate decomposition in order to achieve the practical advantage, namely of reduction of the overall operation time. By considering two non-trivial examples, it is shown that the aforementioned advantage can indeed be achieved in the presented scheme. Our result also provides an important scientific message, i.e., the interplay between “classical” and “quantum” can be helpful in QEC.

## Introduction

Recently, our forefront agenda of quantum computation is to establish efficient quantum error correction (QEC) schemes^1–4^. QEC is applied to correct various (quantum) errors for faithful quantum computation. To accomplish this, most QEC schemes require additional resources such as, ancillary qubits and gates for extracting and correcting the error without altering quantum properties on main computing qubits^5–7^. Such an overall register of qubits, including those for QEC, is usually called a *logical* qubit^4^. Typically, the ability to correct errors can be improved in a recursive way, particularly to achieve fault tolerance^3^. For example, a bundle of logical qubits can be defined as the second level of a logical qubit, hence any higher level of a logical qubit. This is called concatenation scheme^2^. However, this inevitably increases computational and/or time resources *exponentially*. Furthermore, non-transversal gates (e.g., for arbitrary phase rotation, or *T* gates) make the aforementioned problem worse^8^. Thus, it is crucial to solve such a resource-overhead problem toward realization of universal quantum computation (QC).

To solve the problem described above, many studies have been conducted in various perspectives. For example, the quantum circuit for QEC can directly be optimized^6,9–11^. There have been many works on the overhead problem for magic-state distillation^12–18^. Conversion between QEC codes having different transversal gates has also been studied^19,20^. Quite recently, an idea to use different QEC codes in each concatenation has been suggested^21–24^. However, such approaches have focused rather on *quantum*, although many *classical* elements should also be casted for universal QC^25^, as described later.

With such background, we start to design an integrated architecture for universal QC^26–31^, consisting of various working layers, i.e., quantum compiler^32–35^, system organizer^25^, logical and physical qubits. Such an architecture that covers both software and hardware has been commonly used in classical computation^36^. Considering this architecture, we suggested a scheme, named “dynamic concatenation (DC)”, for lower-overhead. The basic idea is to arrange concatenation level, *dynamically*, according to gate operation that the computer is trying to use. This scheme is intuitively understandable: i.e., *with lower-performance* (*or higher-performance*) *gates*, *more* (*or less*) *requirements of concatenation level would be imposed*. Since the gate-performance would strongly depend on the operation to be realized, such an idea is quite natural and reasonable. For example, it is generally harder to implement multi-qubit gates, i.e., correlating gates, than single-qubit gates^37–40^. We derive the effective lower and upper bounds of the gate-decomposition length that allow us to achieve the practical advantage, namely of reduction of the overall operation time. We then apply our DC scheme to the nontrivial examples: quantum Fourier transformation and ground state estimation algorithm on five (logical) qubits. By using Steane code^41^, we show that the aforementioned advantage can indeed be achieved. We believe that our approach will bring forward practical advantages and provide intuition on how classical-quantum interplay can improve the QEC.

## Results

### Integrated QC architecture

**Figure 1:**
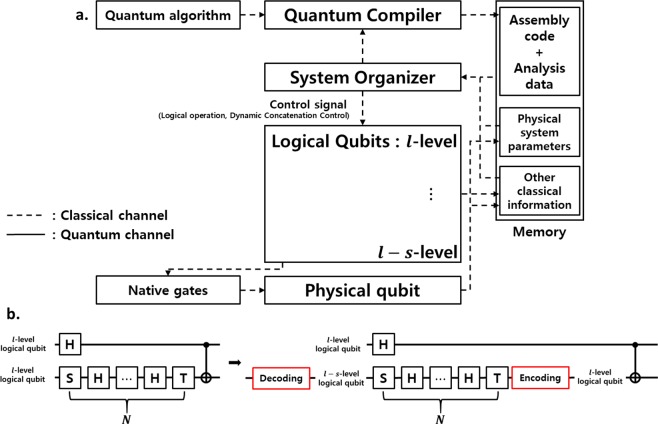
Schematic picture of our integrated QC architecture and dynamic concatenation. (**a**) An integrated QC architecture. The quantum compiler decomposes a quantum algorithm into an assembly code. The assembly code and the maximum tolerable error rate *ε*_*τ*_ are stored in a classical memory. The system organizer manages and controls logical qubits. In particular, it evaluates the concatenation level required to run the algorithm. The block of the logical qubits performs QC according to a set of logical gate operations. The physical-qubit block is responsible for the control of native gates at physical level. (**b**) A simple illustration of our dynamic concatenation (DC) scheme. By using our DC scheme, we can reduce concatenation level of a (logical) qubit from *l* to *l* − *s*. Of course, the additional processes (denoted as red boxes) for decoding and encoding should be adopted to complete the DC and it imposes the extra cost. Nevertheless, it is expected to achieve practical advantage, reduction of the overall operation time, for a sequence within a length, say *N*, of single (logical) qubits. Here, the effective lower and upper bound of *N*, which enable us to achieve the aforementioned advantage, is derived theoretically (see the main text).

We will briefly describe our integrated architecture for QC consisting of the following working layers: (1) quantum compiler, (2) system organizer, (3) logical qubits, and (4) physical qubits (See Fig. 1a). In such an architecture, the process of QC runs as follows. Firstly, let us start with an operation or kernel (e.g., quantum Fourier transform) in a program. The information of the operation is delivered to the first working layer, i.e., quantum compiler. The main role of the quantum compiler is to decompose the delivered operation into a proper set of universal (logical) gates. The sequence of the decomposed gates is called “assembly code.” The quantum compiler also computes the so-called maximum tolerable error rate (MTER) *ε*_*τ*_^34^. Here, note that the error of every decomposed gate should be lower than *ε*_*τ*_. The evaluated *ε*_*τ*_ and assembly code are stored in a classical memory *M*. Then, the system organizer manages the whole system and controls logical qubits using the aforementioned information. The concatenation level required to complete the operation is typically evaluated in this working layer, system organizer. Conventionally, the evaluation of the required concatenation level is made, particularly depending on the lowest-performance gates in the whole decomposed set. However, the evaluation is done group-by-group in the set, i.e., *dynamically*, in our scheme (as described later). The block of logical qubits is responsible for implementing the logical gates assigned by the system organizer. This logical-qubit block also decomposes each logical gate again into native gates implemented at physical hardware level. Here, by “native gate” we mean that the gate is native to the physical hardware^31^. We note that every information used in each working layer are communicated through classical channel ${\mathscr{C}}$, such as, I/O bus. Such an architecture is quite common in classical (and also quantum) computation^36^.

### Dynamic concatenation (DC) scheme

In a typical scheme of QEC, the level of concatenation would be evaluated excessively large sometimes, because the evaluation is made based on the lowest-performance gates in the entire process. This necessarily causes a waste of computational resources. For example, let us consider that we meet a long series of single-qubit (decomposed) gates and a few two-qubit gates in a compilation. Here, noting that in general the single-qubit gates have lower error rates compared to the multi-qubit gates, it seems unreasonable to fix the concatenation levels based on the lowest-performance (mostly, two-qubit) gates. Thus, here we suggest a novel scheme, named dynamic concatenation (DC), that is to apply the concatenation group-by-group of the gates in the middle of the process. We expect that this DC scheme allows us to achieve a practical advantage and can be realized without spending too much extra cost, particularly when a long series, say *N*, of single-qubit gates is encountered in the assembly code.

Then, we derive the range of *N* in which the DC effectively works. Firstly, let us assume that a concatenation level is reduced from *l* to *l* − *s*. Here, *s* is the number of concatenations lowered by the DC. Then, by using the condition that the gate errors should be less then *ε*_*τ*_, we can write the upper bound of *N*, such that

1 $$(\prod _{i=1}^{s}\,{F}_{D}^{(l-i)}{F}_{E}^{(l-i)}){F}_{1Q}^{{(l-s)}^{N}}\ge 1-\frac{{\varepsilon }_{\tau }}{\gamma }$$

where ${F}_{1Q}^{(l-s)}$ is the fidelity of the single-qubit gates at the reduced, i.e., *l* − *s*, concatenation level and the factor *γ* is adopted for more tight condition. ${F}_{D}^{(l-i)}$ and ${F}_{E}^{(l-i)}$ are respectively the fidelity of the decoding and encoding, which have to be taken into account in order to analyze the extra cost of the DC (in terms of the fidelity degradation). This extra cost imposed by the imperfection, i.e, ${F}_{D,E}^{(l-i)} < 1$, of the encoding and/or decoding should not violate the condition related to MTER. To proceed, we then adopt the approximation as

2 $$\prod _{i=1}^{s}\,{F}_{D}^{(l-i)}{F}_{E}^{(l-i)}\simeq {F}_{{\rm{CNOT}}}^{{(l-1)}^{53s}},$$

where ${F}_{{\rm{CNOT}}}^{(l-1)}$ is the fidelity of CNOT gates at *l* − 1 concatenation level. Such an adoption is acceptable, as a product of encoding and decoding fidelities is bounded by ${F}_{{\rm{CNOT}}}^{{(l-1)}^{53}}$. Here, the exponent 53 is made by counting the effective propagation of the error in the whole encoding and decoding circuits (Please see supplementary information). Then, by assuming that ${F}_{1Q}^{(l-s)}\le {F}_{{\rm{CNOT}}}^{(l-1)}$, we can characterize the upper bound of *N* as the following form:

3 $$N\le \lfloor \frac{\mathrm{log}\,(1-\frac{{\varepsilon }_{\tau }}{\gamma })}{\mathrm{log}\,{F}_{1Q}^{(l-s)}}-53\,s\rfloor ,$$

where $\lfloor \cdot \rfloor$ denotes the flooring operation.

Now, we turn to the lower bound of *N*. This lower bound can be obtained by assuming the reduction of the time to complete *N* series of single-qubit gates; i.e.,

4 $$\frac{1}{\prod _{i=1}^{s}\,{F}_{D}^{(l-i)}{F}_{E}^{(l-i)}{F}_{1Q}^{{(l-s)}^{N}}}\,(\sum _{i=1}^{s}\,({T}_{D}^{(l-i)}+{T}_{E}^{(l-i)})+N{T}_{1Q}^{(l-s)}) < N\frac{{T}_{1Q}^{(l)}}{{F}_{1Q}^{{(l)}^{N}}},$$

where *T*_1*Q*_ is the required time for single-qubit gate operation; *T*_*D*_ and *T*_*E*_ are the time to complete the additional decoding and encoding, which are also adopted for the analysis of the extra cost of the DC (in terms of the time delay), together with ${F}_{D}^{(l-i)}$ and ${F}_{E}^{(l-i)}$. Then, by using Eq. (2) and assuming

5 $$\sum _{i=1}^{s}\,({T}_{D}^{(l-i)}+{T}_{E}^{(l-i)})\simeq s({T}_{D}^{(l-\mathrm{1)}}+{T}_{E}^{(l-\mathrm{1)}}),$$

we can arrive at the lower bound of *N* as

6 $$N\ge \lceil (\frac{s({T}_{D}^{(l-1)}+{T}_{E}^{(l-1)})}{{T}_{1Q}^{(l)}})\frac{1}{{F}_{{\rm{CNOT}}}^{{(l-\mathrm{1)}}^{53s}}}\rceil ,$$

where $\lceil \cdot \rceil$ denotes the ceiling operation. Here, we note that the first term $s({T}_{D}^{(l-\mathrm{1)}}+{T}_{E}^{(l-\mathrm{1)}}){T}_{1Q}^{{(l)}^{-1}}$ is dominant, because $1/{F}_{{\rm{CNOT}}}^{{(l-\mathrm{1)}}^{53s}}$ is close to 1. Consequently, we can derive the effective range of *N* enabling the practical advantage, namely of the fidelity enhancement and/or reduction of the operation time:

7 $$\lceil (\frac{s({T}_{D}^{(l-\mathrm{1)}}+{T}_{E}^{(l-\mathrm{1)}})}{{T}_{1Q}^{(l)}})\frac{1}{{F}_{{\rm{CNOT}}}^{{(l-\mathrm{1)}}^{53s}}}\rceil \le N\le \lfloor \frac{\mathrm{log}\,(1-\frac{{\varepsilon }_{\tau }}{\gamma })}{\mathrm{log}\,{F}_{1Q}^{(l-s)}}-53\,s\rfloor .$$

**Figure 2:**
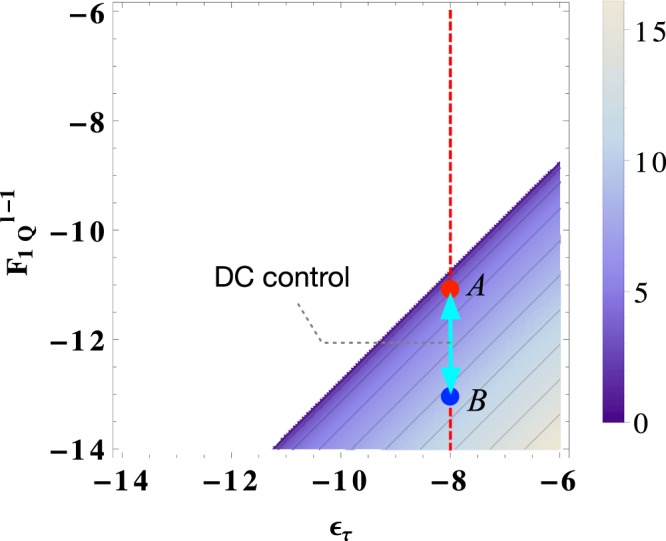
A density-plot of the effective *N* with respect to ${F}_{1Q}^{(l-\mathrm{1)}}$ and ${\varepsilon }_{\tau }$ (on a log-log scale). A colored region indicates the length *N* that allows us to lower the concatenation level, satisfying Eq. (6). This graph also provides a useful intuition about how our DC scheme works. For instance, consider two points: ${F}_{1Q}^{(l)}\simeq {10}^{-13}$ (blue, denoted *B*) and ${F}_{CNOT}^{(l)}\simeq {10}^{-11}$ (red, denoted *A*) in the line of ${\varepsilon }_{\tau }\simeq {10}^{-8}$. The concatenation level is conventionally determined by the lowest performance gates, i.e., the point *A*. However, in our DC scheme, the concatenation can be controlled dynamically between from *A* to *B*, depending on the number *N* of decomposed gates, providing the advantage (see the main text).

Analyzing further, we draw the graph of log *N* with respect to log *ε*_*τ*_ and $\mathrm{log}\,{F}_{1Q}^{(l-s)}$ by using Eq. (7) (see Fig. 2). In particular, we specify the region of $(\mathrm{log}\,{\varepsilon }_{\tau },\,\mathrm{log}\,{F}_{1Q}^{(l-\mathrm{1)}})$ which can bring the above-described advantages by the DC. Such a specification can offer a better intuition of how our DC works. For example, let us consider the hypothetical values of single-qubit and two-qubit gate performances at *l* − 1 concatenation level: ${F}_{1Q}^{(l)}\simeq {10}^{-13}$ (blue point, denoted *B*) and ${F}_{CNOT}^{(l)}\simeq {10}^{-11}$ (red point, denoted *A*). Here, let *ε*_*τ*_ to be $\simeq {10}^{-8}$. In a conventional scheme, the level *l* of the concatenations required to complete a QC has been determined only for the lowest-performance gates, i.e., the point *A*. However, in our DC scheme, the system organizer controls the concatenations dynamically, when a series *N* of single-qubit gates is met in an assembly code and its length *N* is satisfied with Eq. (7). In this case, the concatenation is determined dynamically, moving between the points A and B. This allows the gain *s* to be achieved by our DC scheme.

### Analysis for five-qubit quantum Fourier transform

**Figure 3:**
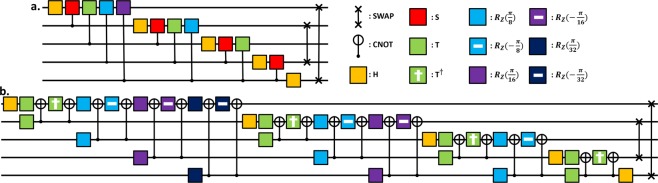
(**a)** *n* = 5 QFT circuit. (Initial states (|0〉) are omitted. Time progresses from left to right.) A total of six controlled-*T* gates and controlled-*R*_*Z*_ gates in this circuit, which generate 10 sequences of the single-qubit gates that cannot be processed in parallel. (**b)** Decomposed QFT circuit. *R*_*Z*_(*φ*) $(\phi =\pm \,\frac{\pi }{8},\pm \,\frac{\pi }{16},\pm \,\frac{\pi }{32})$ will be decomposed into 250 single-qubit gates each in this circuit. Thus there are 18 sequences *R*_*Z*_(*φ*) of the single-qubit gates. However, eight of them are processed in parallel and 10 sequences affect the total processing time.

As an example, we consider five-qubit quantum Fourier transform (QFT). Firstly, we draw a circuit to run QFT at the program level (see Fig. 3a), where the Hadamard *H* and the conditional phase-rotation (i.e., $\frac{\pi }{2}$, $\frac{\pi }{4}$, $\frac{\pi }{8}$, and $\frac{\pi }{16}$) gates are employed. The last two gates are SWAP^42^. Here, we omit the parts of initial states ${|0\rangle }^{\otimes 5}$ and measurements at the end. These operations would be programmed as a set of commands by a user. The quantum compiler decompose these operations into the logical gates, i.e., *H*, *S*, *T*, *R*_*Z*_(*φ*) $(\phi =\pm \,\frac{\pi }{8},\pm \,\frac{\pi }{16},\pm \,\frac{\pi }{32})$, and controlled-NOT (CNOT) gates (as in Fig. 3b), where *R*_*Z*_(*φ*) is the arbitrary *φ*-rotation gate; *S* = *R*_*Z*_ (*π*/2) and *T* = *R*_*Z*_ (*π*/4). Here, we note that *R*_*Z*_(*φ*) is decomposed into, approximately, more than 250 of *H*, *T*, and *S* gates again^35,43^. For simplicity of analysis, we do not consider the decomposition of the last two SWAPs. Actually, SWAP operation would be more primitive, e.g., in a quantum-dot system. The quantum compiler should also evaluate MTER *ε*_*τ*_. In the evaluation, we assume ${\varepsilon }_{\tau }\simeq {10}^{-12}$ and the threshold value ${p}_{th}\simeq 2.7390\times {10}^{-5}$ of Steane code^34,44^. We assume further that the performance of the single-qubit gate is better than that of the two-qubit controlled gate^45,46^. Lastly, we do not take into account the measurement and magic-state usage to calculate the gate operating time. Thus, our calculations has no influence on the generality of the results, because high-level gates require a longer time to create a magic-state^17^ and a higher level of logical qubit also requires more syndrome measurements.

On the basis of the analysis, it is found that three concatenation levels are required to complete the 5-qubit QFT in a conventional QEC. However, in the case of using our DC scheme, only two concatenations are sufficient for 0 < *N* ≤ 91. In particular, we can prove that the overall operation time can be reduced (more than 20 times), such that

8 $${T}_{CC}\simeq 3.5331\times {10}^{-2}\ge {T}_{DC}\simeq 1.4766\times {10}^{-3},$$

where *T*_*CC*_ and *T*_*DC*_ are the operation time for conventional scheme and our DC scheme, respectively. This result is intuitively understandable, i.e., *more* (*or less*) *concatenations are imposed for lower* (*or higher*) *performance gates*, *such as two-qubit controlled gates* (*or single-qubit gates*). Therefore, we expect that such a speed-up will be more conspicuous for large-qubit QFT, which is decomposed into a huge number of single-qubit gates (*H*, *T*, and *S*).

### Analysis for quantum ground state estimation

We then consider the ground state estimation (GSE), which is an algorithm for finding the lowest energy (or state) of a Hamiltonian. The polynomial time quantum GSE runs based on the QFT and quantum phase estimation, where the energies are usually estimated for the number *m* of wave functions (called molecular weight) with *b*-bit of precision. Here, we consider a molecule having *m* = 40 with *b* = 3 bit of precision. Subsequently, we have ${\varepsilon }_{\tau }\simeq 7.0353\times {10}^{-12}$, which is evaluated by ScaffCC compiler^34^. In such a setting, the (logical) gate performance can be calculated on each level of concatenation. The results are listed in Table 1. Based on these results, we can infer that, in a conventional QEC scheme, three concatenations are required to complete quantum GSE, because the error rate of the two-qubit gate cannot reach ${\varepsilon }_{\tau }\simeq 7.0353\times {10}^{-12}$ until level-3 of concatenation. However, if we consider only single-qubit gate, it is sufficient to adopt level-2 of concatenation. Thus, our DC scheme will bring speed-up, preventing waste of the computational time and resource. Actually, our results reveal that we could reduce one-level of concatenation.

**Table 1:** Characteristics of logical gates at a concatenation level 2 and 3.

	Level-2 Concatenation		Level-3 Concatenation	
	Operating Time (sec)	Error Rate	Operating Time (sec)	Error Rate
Single-Qubit Gate	≃1.9977 × 10^−7^	≃4.8665 × 10^−15^	≃1.4009 × 10^−5^	≃8.6481 × 10^−25^
Two-Qubit Gate	≃1.0063 × 10^−7^	≃4.8665 × 10^−11^	≃1.4010 × 10^−5^	≃8.6481 × 10^−17^

Whether a one-qubit gate or a two-qubit gate, there is no significant difference in terms of operating time due to the common error correction process. However, the error rate of the single-qubit gate is about 10^−13^ times lower than *ε*_*τ*_. We can reduce the operating time of the single-qubit gates sequence by reducing this gap through the DC.

## Discussion

We have suggested a novel QEC concatenation scheme to reduce the overall operation time. Our main idea was to evaluate the concatenation, dynamically. The presented scheme was named “dynamic concatenation (DC)” in this sense. The presented scheme was expected to work effectively for a series *N* of decomposed single-qubit (logical) gates, providing the aforementioned advantage. The effective range of *N* was derived theoretically. We then applied our DC scheme to the computation of quantum Fourier transform and quantum ground state estimation on five (logical) qubits. As a result, we explicitly showed the expected advantage. Indeed, such an advantage could be utilized and enabled by the integrated QC architecture that consists of quantum-classical hybridized working layers. In this sense, our work also implies an important scientific message, that is, a proper interplay between “classical” and “quantum” would be very important for the realization of the universal QC. We believe that the presented scheme could be improved more by incorporating other useful schemes.

## Supplementary information

Supplementary Information
