# Supplementary material for: Dynamic Concatenation of Quantum Error Correction in Integrated Quantum Computing Architecture
Source: Sci Rep. 2019 Mar 1;9:3302. doi: 10.1038/s41598-019-39439-0 (PMC6397214; doi:10.1038/s41598-019-39439-0)
Supplement: Supplementary file 1 — Supplementary Information [file 41598_2019_39439_MOESM1_ESM.pdf]

Supplementary Information for "Dynamic Concatenation of Quantum Error Correction in Integrated Quantum Computing Architecture"

Ilkwon Sohn[1], Jeongho Bang[2], and Jun Heo[1,*]

[1]School of Electrical Engineering, Korea University, Seoul, Korea
[2]School of Computational Sciences, Korea Institute for Advanced Study, Seoul 02455, Korea
[*]Correspondence and requests for materials should be addressed to JH (junheo@korea.ac.kr).

ABSTRACT

Supplementary Information for "Dynamic Concatenation of Quantum Error Correction in Integrated Quantum Computing Architecture"

Approximation of criteria for the dynamic concatenation

We show the approximation form of the criteria Eq. (1) for the concatenation-level control of the logical qubits in the main text. In this supplementary information, we will show how the approximation form of criteria was derived.

$$\left\lceil \left(\frac{s(T_D^{(l-1)} + T_E^{(l-1)})}{T_{1Q}^{(l)}} \right) \frac{1}{F_{\text{CNOT}}^{(l-1)^{53s}}} \right\rceil \leq N \leq \left\lfloor \frac{\log\left(1 - \frac{\varepsilon_\tau}{\gamma}\right)}{\log F_{1Q}^{(l-s)}} - 53s \right\rfloor , \tag{1}$$

As mentioned in the main text, when the concatenation level of the logical qubits is lowered, the decrease in the total fidelity should be tolerated. First, we approximate the upper bound Eq. (2) of N.

$$\left(\prod_{i=1}^{s} F_D^{(l-i)} F_E^{(l-i)} \right) F_T^{(l-s)^{N_T}} F_H^{(l-s)^{N_H}} F_S^{(l-s)^{N_S}} \geq 1 - \frac{\varepsilon_\tau}{\gamma}, \tag{2}$$

where $F_D^{(l-i)}$, $F_E^{(l-i)}$ are fidelity of encoding decoding circuit, $F_T^{(l-s)}$, $F_H^{(l-s)}$, $F_S^{(l-s)}$ are the performance of T, H, S gate at $l-s$ level and N_T, N_H, N_S are the number of T, H, S gate. DC-level s is the number of concatenations lowered by our scheme. ε_τ is the maximum tolerable error rate obtained by the quantum compiler through algorithm analysis. All operations in computing should have an error rate lower than ε_τ. We calibrated ε_τ to a factor of γ to get a more conservative approach to the results(Here, we set $\gamma = 10$).

The performance of single-qubit gates is approximated to T gate because it is expected to have the worst performance among single-qubit gates. Hereafter, we denote $F_T^{(l-s)}$ as $F_{1Q}^{(l-s)}$ which means the fidelity of a singe-qubit gate. Also, the sum of N_T, N_H, and N_S is denoted by N, which means the length of a single qubit gates sequence.

The product of the en/decoding circuits' fidelities at each level can be approximated by the product of the highest level en/decoding circuits' fidelities. Note that these en/decoding circuits for Steane code are not fault-tolerant.

$$\prod_{i=1}^{s} F_D^{(l-i)} F_E^{(l-i)} \approx \left(F_D^{(l-1)} F_E^{(l-1)} \right)^s, \tag{3}$$

Therefore, Eq. 2 is approximated as follows.

$$\left(F_D^{(l-1)} F_E^{(l-1)} \right)^s F_{1Q}^{(l-s)^N} \geq 1 - \frac{\varepsilon_\tau}{10}, \tag{4}$$

In most cases, the en/decoding circuits consist of Hadamard gates and CNOT gates. The fidelities of the en/decoding circuits are determined by the fidelities of Hadamard gates and CNOT gates and it can be approximated with the worst

one. We assume that the performance of a two-qubit gate is worse than that of a single-qubit gate, so each fidelity can be approximated as follows.

$$\begin{aligned} F_D^{(l-1)} &\simeq F_{\mathrm{CNOT}}^{(l-1)\,p_D}, \\ F_E^{(l-1)} &\simeq F_H^{(l-1)\,p_{E,H}} F_{\mathrm{CNOT}}^{(l-1)\,p_{E,\mathrm{CNOT}}} \approx F_{\mathrm{CNOT}}^{(l-1)\,p_E}, \end{aligned} \tag{5}$$

where p_D, $p_{E,H}$, and $p_{E,\mathrm{CNOT}}$ are factors related to the fidelities of the encoding and decoding circuits and p_E is sum of $p_{E,H}$ and $p_{E,CNOT}$. There are many ways to obtain these values, the method used in the main text is explained in chapter 2. Fidelities of en/decoding circuits can be approximated about $F_{\mathrm{CNOT}}^{(l-1)}$

$$(F_D^{(l-1)} F_E^{(l-1)})^s \approx F_{\mathrm{CNOT}}^{(l-1)\,s\cdot p_{E,D}}, \tag{6}$$

where $p_{E,D}$ is sum of p_D and p_E. Hereafter, we set $p_{E,D} = 53$ Then Eq. (4) is approximated as follows.

$$F_{\mathrm{CNOT}}^{(l-1)\,53s} F_{1Q}^{(l-s)\,N} \geq 1 - \frac{\varepsilon_\tau}{10}, \tag{7}$$

We assume that $F_{1Q}^{(l-s)}$ is worse than $F_{\mathrm{CNOT}}^{(l-1)}$ and the formula can be approximated as follows.

$$F_{1Q}^{(l-s)\,N+53s} \geq 1 - \frac{\varepsilon_\tau}{10}, \tag{8}$$

Then we can get the upper bound of Eq. (9) by taking logarithm on both sides of the Eq. (8).

$$N \leq \left\lfloor \frac{\log\left(1 - \frac{\varepsilon_\tau}{\gamma}\right)}{\log F_{1Q}^{(l-s)}} - 53s \right\rfloor, \tag{9}$$

Now we approximate the lower bound Eq. (10) of N.

$$\frac{1}{\prod_{i=1}^{s} F_D^{(l-i)} F_E^{(l-i)} F_{1Q}^{(l-s)\,N}} \left(\sum_{i=1}^{s} \left(T_D^{(l-i)} + T_E^{(l-i)} \right) + N T_{1Q}^{(l-s)} \right) < N \frac{T_{1Q}^{(l)}}{F_{1Q}^{(l)\,N}}, \tag{10}$$

where $T_D^{(l-i)}$ and $T_E^{(l-i)}$ are $l-i$ level encoding, decoding time and $T_{1Q}^{(l)}$ is l level single-qubit gate operating time. Eq. 10 can be rewritten as:

$$\frac{\sum_{i=1}^{s} \left(T_D^{(l-i)} + T_E^{(l-i)} \right)}{\left(\frac{F_{1Q}^{(l-s)\,N}}{F_{1Q}^{(l)\,N}} \prod_{i=1}^{s} F_D^{(l-i)} F_E^{(l-i)} T_{1Q}^{(l)} \right) - T_{1Q}^{(l-s)}} \leq N, \tag{11}$$

The product of the en/decoding circuits' fidelities can be approximated by the product of the highest level en/decoding circuits' fidelities. Since the performance of the single-qubit gates at any level is close to one, we will approximate $\frac{F_{1Q}^{(l-s)\,N}}{F_{1Q}^{(l)\,N}}$ by one. Then Eq. (11) can be rewritten as:

$$\left\lceil \frac{\sum_{i=1}^{s} \left(T_D^{(l-i)} + T_E^{(l-i)} \right)}{(F_D^{(l-1)} F_E^{(l-1)})^s T_{1Q}^{(l)} - T_{1Q}^{(l-s)}} \right\rceil \leq N, \tag{12}$$

Because the operating time at the highest level will be the longest, we approximate $\sum_{i=1}^{s} \left(T_D^{(l-i)} + T_E^{(l-i)} \right)$ by $s \left(T_D^{(l-1)} + T_E^{(l-1)} \right)$

$$\left\lceil \frac{s \left(T_D^{(l-1)} + T_E^{(l-1)} \right)}{(F_D^{(l-1)} F_E^{(l-1)})^s T_{1Q}^{(l)} - T_{1Q}^{(l-s)}} \right\rceil \leq N, \tag{13}$$

$(F_D^{(l-1)} F_E^{(l-1)})^s$ can be approximated by $(F_{\mathrm{CNOT}}^{(l-1)})^{53s}$. Since $(F_D^{(l-1)} F_E^{(l-1)})^s T_{1Q}^{(l)}$ is large enough, $T_{1Q}^{(l-s)}$ can be ignored. Therefore we can obtain the final form of the lower bound.

$$N \geq \left\lceil \left(\frac{s(T_D^{(l-1)} + T_E^{(l-1)})}{T_{1Q}^{(l)}} \right) \frac{1}{F_{\mathrm{CNOT}}^{(l-1)\,53s}} \right\rceil, \tag{14}$$

Fidelity factor of en/decoding circuit

We use a factor $p_{E,D}$ in Eq. (9) and its value is related to the fidelities of the en/decoding circuits. We assume that the whole fidelity will be degraded as the gate fidelity by multiplying the fidelity of the gate to the whole fidelity. We will briefly show the en/decoding circuits of the Steane code we used and the $p_{E,D}$ obtained from it. Each circuit is not fault-tolerant, but it is assumed that each qubit is a logical qubit so it can tolerate some errors in the en/decoding process.

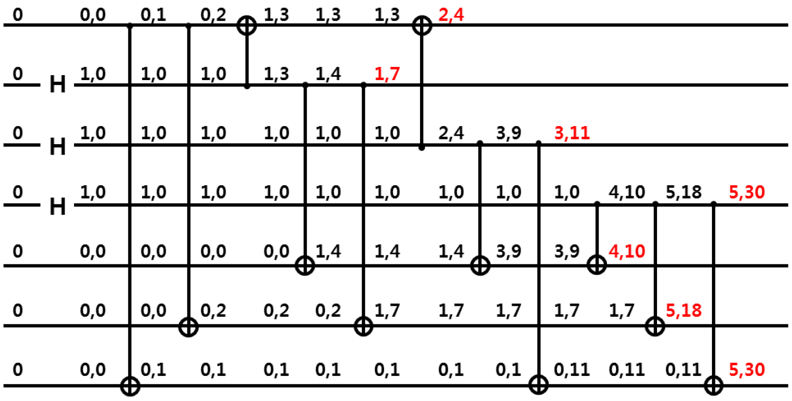

Figure 1. Encoding circuit of Steane code and its p_E value(Initial states are omitted. Time progresses from left to right.) We consider that the influence of each gate on fidelity propagates through a CNOT gate. $p_{E,H}$ and $p_{E,\mathrm{CNOT}}$ are 5 and 30, respectively. Finally, the value of p_E is 35.

In fig. 1, the number before the comma is $p_{E,H}$ associated with the single-qubit gates and the number after it is $p_{E,\mathrm{CNOT}}$ associated with the two-qubit gates. The red number represents the final p value of that qubit line at the very end. We define the largest number of the red numbers as the p value of the whole circuit. In the case of fig. 1, $p_{E,H}$ is 5, $p_{E,\mathrm{CNOT}}$ is 30, and p_E is 35.

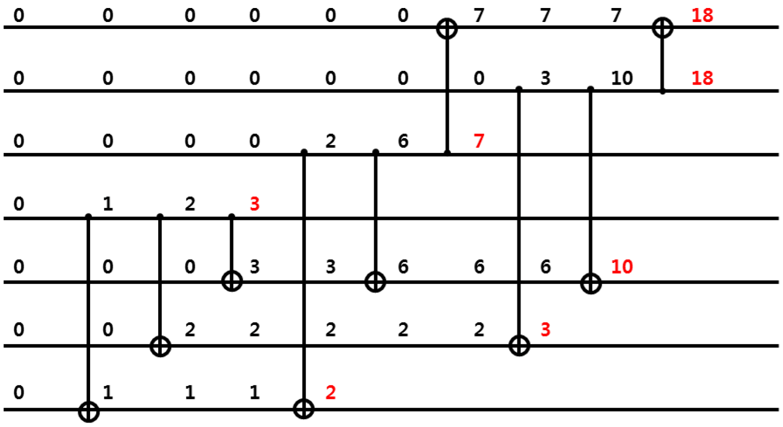

Figure 2. Decoding circuit of Steane code and its p_D value(Initial states are omitted. Time progresses from left to right.) We also consider that the influence of each gate on the fidelity is propagated through the CNOT gate in the decoding circuit. We do not consider the correction through the ancilla qubit measurement at the final stage, thus we omit the several CNOT and Hadarmard gates in this circuit. Finally, the value of p_D is 18.

In the case of fig. 2, p_D is 18. Then the $p_{E,D}$ is obtained by adding p_E and p_D, in this case 53.

Estimation of N for the dynamic concatenation

With Eq. (9), we have calculated the example of N with the certain ε_τ, $F_{1Q}^{(l-1)}$ when $s = 1$ and $p_{E,D} = 53$. Fig. 3. and Table 1. represent N with these parameters.

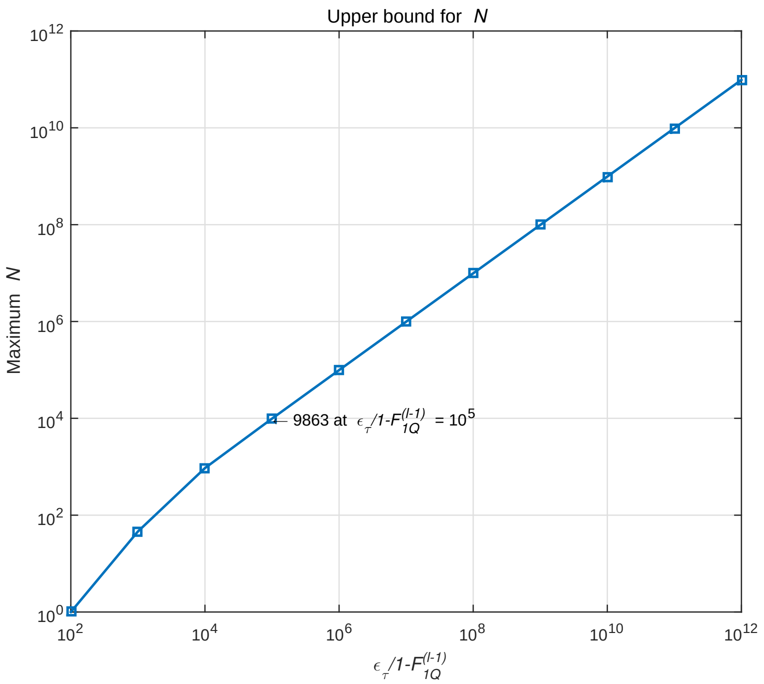

Figure 3. Logarithmic scale graph for upper bound on N. This graph is plotted N against $\varepsilon_\tau/(1-F_{1Q}^{(l-1)})$ on Logarithmic scales by averaging the values in Table 1. As $\varepsilon_\tau/(1-F_{1Q}^{(l-1)})$ exponentially increases, N also exponentially increases. When $\varepsilon_\tau/(1-F_{1Q}^{(l-1)})$ is 10^5, a single-qubit gate sequence with a length of 9863 can be operated with the concatenation level being one step lower.

We can check that N is almost the same depending on the ratio of ε_τ to $1-F_{1Q}^{(l-1)}$. If $1-F_{1Q}^{(l-1)}$ and ε_τ differ by 10^{-5} times, it can be seen that N is close to 10,000, and the above values are expected that there will be no meaning in a practical case.

In such a situation, we can lower the concatenation level further, rather than use the meaninglessly long N. Table 2. represents how the concatenation level can be lowered for $\varepsilon_\tau = 1.0\times10^{-6}$, $1.0\times10^{-10} \le 1-F_{1Q}^{(l-1)} \le 1.0\times10^{-6}$.

The DC may seem impractical because we only deal with too large N or s. At the logical qubit level, however, we can get sufficient gain by lowering the concatenation level by one or two steps. This simple example will provide how much gain can be achieved by lowering the concatenation level one step. When $R_Z(\frac{\pi}{8})$ is decomposed and executed, $R_Z(\frac{\pi}{8})$ can be decomposed into approximately 250 other single gates as follows.
HTHTSHTHTSHTSHTHTHTHTHTSHTSHTSHTHTSHTSHTSHTSHTHTSHTSHTSHTHTHTSHTSHTSHTHTHTH
THTHTSHTSHTHTHTSHTSHTSHTSHTSHTSHTSHTSHTHTHTHTSHTSHTHTSHTSHTHTHTHTSHTSHTSHTH
TSHTSHTHTHTHTHTSHTHTSHTHTHTSHTSHTSHTSHTHTHTSHTHTSHTSHTHTHTHTSHTSHTHTSHTSHTH
TSHTSHTSHTSHTHTSHTHTHTSHTH.

If the concatenation level can be lowered by one, we can obtain a gain of approximately 22 times in the operating time with the condition of table 1. in main text.

$1-F_{1Q}^{(l-1)}$ \ ε_τ	1.0×10^{-4}	1.0×10^{-5}	1.0×10^{-6}	1.0×10^{-7}	1.0×10^{-8}	1.0×10^{-9}	1.0×10^{-10}
1.0×10^{-6}	-43.0	-	-	-	-	-	-
1.0×10^{-7}	47.0	-43.0	-	-	-	-	-
1.0×10^{-8}	947.0	47.0	-43.0	-	-	-	-
1.0×10^{-9}	9947.1	947.0	47.0	-43.0	-	-	-
1.0×10^{-10}	99947.5	9947.0	947.0	47.0	-43.0	-	-
1.0×10^{-11}	999951.9	99947.0	9947.0	947.0	47.0	-43.0	-
1.0×10^{-12}	10000218.2	999969.6	99949.2	9947.2	947.0	47.0	-43.0
1.0×10^{-13}	99969362.0	9996843.5	999636.2	99915.9	9943.9	946.7	47.0
1.0×10^{-14}	1000804868.2	10007998.8	10007946.7	1000746.9	100027.0	9955.0	947.8
1.0×10^{-15}	10008049159.2	1000800364.6	100079943.7	10007946.3	1000746.9	100027.0	9955.0
1.0×10^{-16}	900724428857.0	9007203705.6	900719917.0	90071940.5	9007146.0	900667.0	90019.0

$1-F_{1Q}^{(l-1)}$ \ ε_τ	1.0×10^{-11}	1.0×10^{-12}	1.0×10^{-13}
1.0×10^{-6}	-	-	-
1.0×10^{-7}	-	-	-
1.0×10^{-8}	-	-	-
1.0×10^{-9}	-	-	-
1.0×10^{-10}	-	-	-
1.0×10^{-11}	-	-	-
1.0×10^{-12}	-	-	-
1.0×10^{-13}	-43.0	-	-
1.0×10^{-14}	47.1	-43.0	-
1.0×10^{-15}	947.8	47.1	-43.0
1.0×10^{-16}	8954.0	848.0	37.0

Table 1. Upper bound for the length of the sequence N when $s=1$. (Case of $1.0\times10^{-13}\le\varepsilon_\tau\le1.0\times10^{-4}$ and $1.0\times10^{-16}\le1-F_{1Q}^{(l-1)}\le1.0\times10^{-6}$). This table represents the upper bound of N according to $1-F_{1Q}^{(l-1)}$ and ε_τ of the algorithm to be performed when the concatenation level is lowered by one step. We can check that the DCC is possible if $1-F_{1Q}^{(l-1)}$ is 10^{-3} times smaller than ε_τ. As mentioned above, this is the maximum N when the concatenation level is lowered by one step. Thus, by lowering the concatenation level further, we can get more reasonable N and s.

	$1-F_{1Q}^{(l-1)}$	s_{max}	$N\ (s_{max})$
$\varepsilon_\tau =$ 1.0000×10^{-6}	1.0000×10^{-6}	0	0
	1.0000×10^{-7}	0	0
	1.0000×10^{-8}	1	47
	1.0000×10^{-9}	18	46
	1.0000×10^{-10}	188	36

Table 2. Upper bound for lowering level s. s_{max} represents the step of concatenation level that can be maximally lowered and $N\ (s_{max})$ represents the upper bound for N at s_{max}. s_{max} become larger as $F_{1Q}^{(l-1)}$ improves, but s is limited by the maximum concatenation level. Therefore it may be not realistic to lower the dozens steps of concatenation level.
